# Hilar lymphadenopathy, development of tubulointerstitial nephritis, and dense deposit disease following Pfizer-BioNTech COVID-19 vaccination

**DOI:** 10.1007/s13730-022-00762-7

**Published:** 2022-12-13

**Authors:** Hironori Nakamura, Michiko Ueda, Mariko Anayama, Mutsuki Makino, Yasushi Makino

**Affiliations:** 1 Department of Nephrology, Shinonoi General Hospital, 666-1 Ai Shinonoi, Nagano, Japan; 2 Department of Pathology, Shinonoi General Hospital, Nagano, Japan

**Keywords:** Alternative pathway, Complement, Glomerulonephritis, Allergy

## Abstract

Despite the reports on glomerulonephritis associated with COVID-19 mRNA vaccines, no study has reported about the dense deposit disease (DDD). Here, we present a case of hilar lymphadenopathy after the COVID-19 mRNA vaccination, following which the patient developed tubulointerstitial nephritis (TIN) and DDD. A 74-year-old man received his second dose of mRNA vaccine, and on the next day, he developed fever, urticaria, and dyspnea. On further examination, he had pleural effusion and right hilar lymphadenopathies, which were improved with conservative therapy. After 48 days of the second vaccination, he developed renal dysfunction and new-onset hematuria. Light microscopy findings by renal biopsy revealed apparent mesangial cell proliferation, increased mesangial matrix in the glomeruli, and diffuse inflammatory cell infiltration in the interstitium. Immunofluorescence analysis revealed 1 + positive results for IgG and IgM, negative results for IgA, and 2 + positive results for C3 with a garland pattern on the capillary walls. Electron microscopy revealed that severe cell proliferation in the capillary rumen, and continuous, thickened, and highly dark-stained spotty dense deposits in the glomerular basement membrane; and noncontinuous spotty dense deposits in the tubular basement membrane. Based on the decrease in C3 and pathological findings, TIN accompanied with DDD was diagnosed. The mRNA vaccine might have contributed to the development of lymphadenopathies, TIN, and DDD in this case. Moreover, TIN and DDD might be associated with the activated alternative pathway induced by the mRNA vaccine.

## Introduction

Antineutrophil cytoplasmic antibody (ANCA)-associated vasculitis [1], minimal change disease, antiglomerular basement membrane (anti-GBM) antibody disease [2, 3], IgA nephropathy [3, 4], and tubulointerstitial nephritis (TIN) [5], which are linked to COVID-19 vaccination, have recently been reported in a growing literature on glomerulonephritis. Complement activation-related pseudoallergy (CARPA) is now recognized as the underlying cause of hypersensitivity reactions to various drugs, such as monoclonal antibody (e.g., rituximab), liposome-encapsulated products (doxil or ambisome), and micellized anticancer drug (paclitaxel) [6]. Here, we present a case of a patient who developed hilar lymphadenopathy, TIN, and dense deposit disease (DDD) following mRNA vaccination.

## Case report

**Fig. 1:**
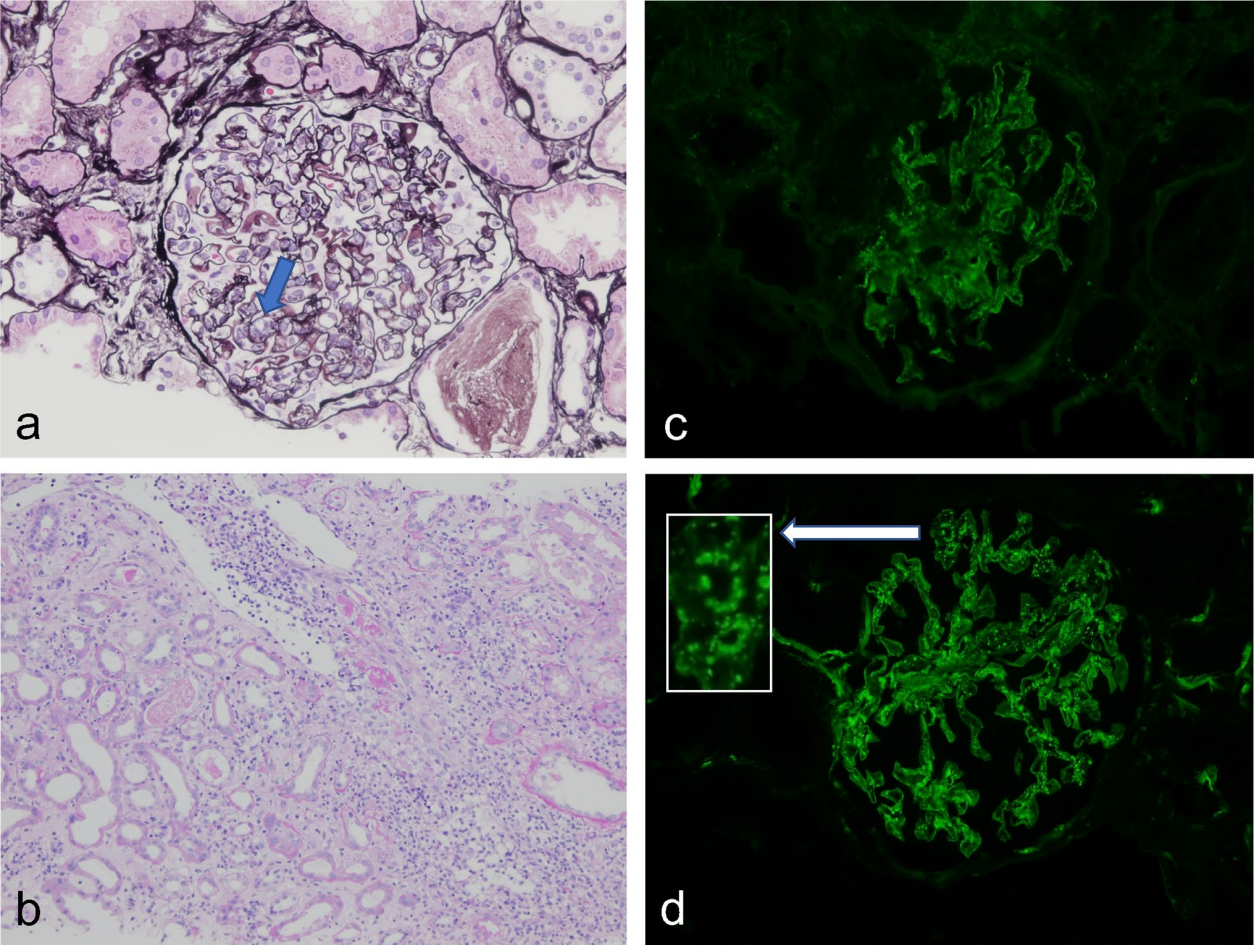
Pathological findings by renal biopsy: light microscopy (**a** and **b**); immunofluorescence for IgG (**c**) and for C3 (**d**). Periodic acid-methenamine silver staining revealed focal endocapillary proliferation (a, arrow) in one glomerulus. Periodic acid-Schiff staining revealed massive and diffuse inflammatory cell infiltration in the interstitium. The majority of infiltratory cells were lymphocytes and plasma cells. A few eosinophilic cells were seen (**b**). Immunofluorescence analysis revealed 1 + positive results for IgG (**c**) and 2 + positive results for C3 on the capillary walls and mesangium area (**d**). A garland pattern is magnified in top left rectangle (**d**)

**Fig. 2:**
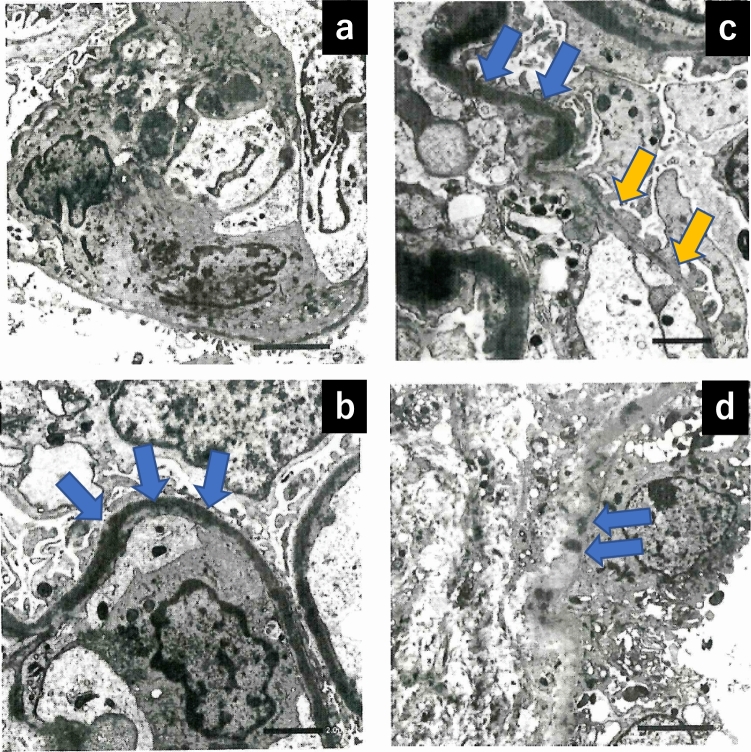
Electron microscopy revealed severe glomerular cell proliferation (**a**) and continuous and dark-stained spotty dense deposits (blue arrows) in the glomerular basement membrane (**b**); the severe thickness of the basement membrane (blue arrows) with dense deposits and the mild thickness of the basement membrane (yellow arrows) with small amounts of dense deposits coexisted (**c**). Noncontinuous spotty dense deposits in the tubular basement membrane (**d**)

A 74-year-old man with a past medical history of hyperlipidemia received his second dose of the Pfizer-BioNTech COVID-19.vaccine (Pfizer, Inc. Philadelphia, PA) on his left upper arm on June 29, 2021. On the next day, he developed fever and urticaria on the trunk and extremities. He took 120 mg of loxoprofen sodium hydrate per day for 7 days. On July 10, 2021, he visited our hospital for gradually deteriorating dyspnea. The levels of serum creatinine (sCr) and C-reactive protein were 0.75 mg/dL and 15.0 mg/dL, respectively, and urinalysis revealed no proteinuria or red blood cells. He presented with right pleural effusion and right hilar lymphadenopathies, which indicated the presence of malignant lesions. The repeated cytological findings of pleural effusion by cellblock demonstrated a reactive pattern, and fine-needle aspiration of an enlarged lymph node revealed no evidence of malignancy; thus, lung carcinoma was denied. The level of C-reactive protein was reduced to the normal range, and lymphadenopathies gradually regressed with the administration of ampicillin/sulbactam for 10 days. After 48 days of the second vaccination, his sCr level increased to 1.31 mg/dL, and new-onset hematuria was observed. Serological tests for ANCA, anti-SSA/Ro, anti-SSB/Ro, anti-DNA, and anti-GBM antibodies were negative. There was no elevation in IgG4, angiotensin-converting enzyme, or anti-streptolysin O titer. A decrease in C3 (50.1 mg/dL; normal range, 73–138 mg/dL) along with the elevated titer of antiScl-70 antibody, anti-cardiolipin antibody, and anti-CLβ2 GP1 antibodies are reported in Table 1. Renal biopsy light microscopy findings revealed 15 glomeruli; of these, 3 were global sclerosis. Periodic acid-methenamine silver staining (PAM) revealed mild mesangial cell proliferation with focal endocapillary proliferation (Fig. 1a; arrow head) in one glomerulus. In addition, both irregularity of the glomerular basement membrane and a double contour were seen segmentally (Fig. 1a). Periodic acid-Schiff staining revealed massive and diffuse inflammatory cell infiltration in the interstitium. The majority of infiltrating cells were lymphocytes and plasma cells. However, a few eosinophilic cells were seen (Fig. 1b). No necrotizing lesions or vasculitis were observed. Immunofluorescence analysis revealed 1 + positive results for IgG (Fig. 1c) and IgM, negative results for IgA, and 2 + positive results for C3 on the capillary and mesangium area (Fig. 1d). A garland pattern was observed (magnified in the top left rectangle in Fig. 1d). Electron microscopy revealed severe glomerular cell proliferation (Fig. 2a); and continuous, thickened, and highly dark-stained spotty dense deposits in the glomerular basement membrane (Fig. 2b; blue arrows). Furthermore, the severe thickness of the basement membrane (Fig. 2c; blue arrows) with dense deposits and the mild thickness of the basement membrane (Fig. 2c; yellow arrows) with small amounts of dense deposits coexisted. The findings of noncontinuous spotty dense deposits (Fig. 2d; blue arrows) in the tubular basement membrane were also observed. The patient did not present with ocular lesions. The drug lymphocyte stimulation test for loxoprofen sodium hydrate, ampicillin/sulbactam, and atorvastatin was negative. To distinguish DDD from monoclonal immunoglobulin deposition disease, immune histochemical staining was carried out using paraffin sections. Both kappa and lambda were found to be positive in glomeruli, but there was no discernible difference between the two light chains. Finally, both serum and urine electrophoresis results indicated the absence of M protein.

**Table 1:** Laboratory date on renal biopsy

white blood cell (/µL)	4600	sodium (mEq/L)	141	ANA	< 40	pH	7.41	
red blood cell (/µL)	376 × 10^4^	potassium (mEq/L)	3.2	IC-c1q (µg/mL)	< 1.5	PCO_2_ (mmHg)	47.7	mmHg
hemoglobin (g/dL)	10.7	calcium (mg/dL)	8.3	anti-DNA (IU/mL)	< 2.0	PO_2_ (mmHg)	25.8	mmHg
hematocrit (%)	32.3	phosphate (mg/dL)	3.0	anti-RNP (U/mL)	< 2.0	lactate (mg/dL)	7	mg/dL
platelet (/µL)	19.2 × 10^4^	creatine phosphokinase (U/L)	122	anti-Sm (U/mL)	< 1.0	HCO_3_	29.5	mmol/L
total protein (g/dL)	6.2	c-reactive protein (mg/dL)	1.14	anti-SSA/Ro (U/mL)	< 1.0			
albumin (g/dL)	3	IgG (mg/dL)	1818	anti-SSB/La (U/mL)	< 1.0	urine-specific gravity	1.011	
total bilirubin (mg/dL)	0.8	IgA (mg/dL)	232	anti-Scl-70 (u/mL)	119	protein	2 +	
aspartate aminotransferase (U/L)	22	IgM (mg/dL)	76	anti-CLβ2GP1 (U/mL)	4.9	occult blood	3 +	
alanine aminotransferase (U/L)	11	IgE	< 25	anti-cardiolipin antibody IgG (U/mL)	16	RBC (/HPF)	100	/HPF
lactate dehydrogenase (U/L)	245	C3 (mg/dL)	50.1	PR3-ANCA (U/mL)	< 1.0	WBC (/HPF)	5–9	/HPF
blood urea nitrogen (mg/dL)	14	C4 (mg/dL)	20.1	MPO-ANCA (U/mL)	< 1.0	N-acetyl-β-D-glucosaminidase (IU/L)	19.4	IU/L
creatinine (mg/dL)	1.28	CH50 (CH50/mL)	29.8	anti-GBM (U/mL)	< 2.0	myoglobin (ng/mL)	84	ng/mL
		IgG4	44.5	cryoglobulin	–

Although, this case presented with several atypical findings, TIN accompanied with DDD was diagnosed based on serological and pathological findings. Prednisolone (50 mg; 0.8 mg/kg) administration was started to treat TIN. At 7 weeks after the renal biopsy, his C3 level returned to the normal range, and the patient’s renal dysfunction and urinary findings gradually improved (Table 2).

**Table 2:** Changes in serum creatine, serum C3, and urinary data after renal biopsy

Weeks after renal biopsy	0	1	2	4	6	7	9	11	13	17
Serum creatinine (0.65–1.07 mg/dL)	1.28	1.62	1.98	1.47	1.53	1.37	1.47	1.41	1.23	1.15
Serum C3 (73–138 mg/dL)	50.1	67.5	66.1	58.5	70.2	76.7	81.0	na	na	na
Urinary NAG	19.4	na	na	7.0	6.3	na	10.1	na	na	9.7
Proteinuria (g/gCr)	0.40	0.29	±	–	–	–	–	–	–	–
Urinary red blood cell (/HPF	> 100	> 100	> 100	50–99	20–29	10−19	50−99	20−29	10−19	10−19

NAG, N-acetyl-β-D-glucosaminidase

*na* not available, *HPF* high-power field

## Discussion

We presented a case of a patient who developed reactive lymphadenopathy, TIN, and DDD, which was believed to be caused by strong complement amplification. Because both coincidental onset with TIN and DDD following an acute allergic response that occurred approximately before 7 weeks led us to believe that each event or disease can be associated with COVID-19 mRNA vaccination via an inflammatory or immunological response following mRNA vaccination. Although TIN caused by nonsteroidal anti-inflammatory drugs and antibiotics cannot be completely ruled out, this is a unique case as it is inconsistent with any known disease entities or clinical course.

Post-vaccination ipsilateral lymphadenopathy typically occurs in readily accessible sites, such as the cervical, axillary and supraclavicular lymph nodes [7]. In the present case, lymphadenopathy occurred on the contralateral side, which mimics lung carcinoma and compresses the intrabrachial duct causing dyspnea; this suggested the involvement of a severe immunological reaction. Although the enlarged lymph nodes degenerated after antibiotic treatment, they might be attributed to acute allergic reactions due to the mRNA vaccine rather than infections or malignancies, based on the results of several clinical tests.

DDD is associated with the deposition of complement C3 in the glomeruli and is thought to result from uncontrolled activation of an alternative signaling pathway [8]. No study has reported DDD associated with mRNA vaccination. IF revealed that IgG staining can be a slightly stronger or C3 can be a slightly weaker marker for the diagnosis of DDD in the present case. Although we could not properly explain these pathological findings, we hypothesized that the duration from the onset of this disease and/or vaccination-induced mechanisms might be related to the atypical findings.

Recently, several cases of TIN after mRNA vaccination have been reported [5]. In a mice model, Turnberg et al. demonstrated that the activation of the alternative pathway rather than classical pathway contributed to glomerular and tubulointerstitial damage [9]. Mira FS et al. [5] revealed that positive lymphocyte transformation test findings for polyethylene glycol and vaccine solution indicated T-cell involvement, which could represent a T-cell-mediated injury. Consequently, aberrant innate and acquired immune responses could be involved in the onset of interstitial nephritis [10, 11].

The SARS-CoV-2 spike protein binds to heparan sulfate on nucleated cells and enhances the complement’s alternative pathway by interfering with the binding of complement factor H, an inhibitor of the alternative pathway [12]. Adverse reactions to vaccines might develop because of the interaction between the vaccinated subject’s susceptibility and various vaccine components. The molecular resemblance between certain pathogenic elements in the vaccine and specific human proteins has been suggested as one of the mechanisms behind these reactions. This resemblance may cause immune cross reactivity, in which the immune system’s reaction to pathogenic antigens destroys similar human proteins, resulting in an autoimmune disease [10]. Although the clinical significance was unknown, the positivity for the two types of antiphospholipid antibodies and decrease in C3 suggest dysregulations of the alternative pathway of complements in this case. An alternative pathway of complement activation may explain many clinical manifestations including microangiopathy, thrombocytopenia, renal injury, and thrombophilia in patients with COVID-19. These are also observed in other complement-driven diseases such as atypical hemolytic uremic syndrome and catastrophic antiphospholipid antibody syndrome [12].

With the C3a and C5a anaphylatoxins binding to mast cells in CARPA syndrome, it has recently been recognized that several modern-day therapeutic molecules may activate complement via a non-IgE mediated mechanism, triggering the release of several vasoactive mediators that cause the clinical features associated with hypersensitivity reactions [6, 13]. Reactive lymphadenopathy, TIN, and DDD may be influenced by such mechanisms. In the present case, hematuria was observed after 7 weeks of vaccination. While the serum creatinine level gradually increased from 0.75 to 1.28 mg/dL over 7 weeks, the urine test was not performed until the day of the case report. Thus, we think that the virtual onset can be much earlier.

In conclusion, COVID-19 vaccination might contribute to the development of hilar lymphadenopathy, TIN and DDD in a patient following mRNA vaccination. Moreover, DDD and TIN might be associated with activated alternative pathway induced by COVID-19 mRNA vaccination.
